# A software tool for the analysis of neuronal morphology data

**DOI:** 10.1186/1755-7682-7-6

**Published:** 2014-02-17

**Authors:** Julia Ledderose, Luis Sención, Humberto Salgado, Oscar Arias-Carrión, Mario Treviño

**Affiliations:** 1 Institute of Biochemistry, Charité Universitätsmedizin Berlin, Berlin, Germany; 2 Departamento de Neurociencias, Centro de Investigaciones Regionales "Dr. Hideyo Noguchi", Universidad Autónoma de Yucatán, Yucatán, México; 3 Unidad de Trastornos del Movimiento y Sueño (TMS), Hospital General Dr. Manuel Gea González, México D.F., México; 4 Instituto de Neurociencias, Universidad de Guadalajara, Guadalajara, México

**Keywords:** Neurons, Dendrites, Morphology, Tortuosity, Simulations

## Abstract

Anatomy plays a fundamental role in supporting and shaping nervous system activity. The remarkable progress of computer processing power within the last two decades has enabled the generation of electronic databases of complete three-dimensional (3D) dendritic and axonal morphology for neuroanatomical studies. Several laboratories are freely posting their reconstructions online after result publication *v.gr.* NeuroMorpho.Org (*Nat Rev Neurosci***7:**318–324, 2006). These neuroanatomical archives represent a crucial resource to explore the relationship between structure and function in the brain (*Front Neurosci***6:**49, 2012). However, such 'Cartesian’ descriptions bear little intuitive information for neuroscientists. Here, we developed a simple prototype of a MATLAB-based software tool to quantitatively describe the 3D neuronal structures from public repositories. The program imports neuronal reconstructions and quantifies statistical distributions of basic morphological parameters such as branch length, tortuosity, branch's genealogy and bifurcation angles. Using these morphological distributions, our algorithm can generate a set of virtual neurons readily usable for network simulations.

## Results and discussion

Electronic databases of complete 3D dendrites constitute a valuable tool to explore the morphological structure of single neurons [1]. The data acquisition of those structures comprises a multi-step process from tissue collection and staining to the extraction of neuronal structural information via a variety of imaging techniques. To date, the majority of dendritic and axonal morphology reconstructions are based on bright-field microscopy mainly because of its broad compatibility with histological staining methods [2]. Digital tracing of neuronal morphology converts large amounts of imaging information into a simple and compact representation which can be easily visualized, quantified, archived, and shared [3], thus maximizing the opportunity to exploit the full potential of unrestricted morphometric analyses [1,4].

There are multiple ways to digitize neuronal morphology once it has been visualized by optical microscopy. One effective way to describe the treelike branching of axons and dendrites can be achieved by using a sequence of interconnected cylinders. In this 'vector' representation, each uniform segment in the arbor can be characterized by five values, consisting of the three Euclidean 'x', 'y' and 'z' coordinates, a diameter of its ending location, and the identity of the 'parent' segment from which each new segment originates. Thus, by definition, compartments are segments represented as cylinders with a given diameter and the coordinates of the extreme points. Branches are formed with one or more compartments between the soma, the bifurcations, and the tips. Bifurcations are defined as the points where a branch splits into 'daughter' branches.

This 'Cartesian' description of neuronal structures constitutes a complete mapping of dendritic morphology but bears little intuitive information. To extract quantitative measures of neuronal morphology, we developed a software tool written in MATLAB (MATLAB R2012a, MathWorks, Inc.) that reads these 3D dendritic reconstructions and computes morphological parameters from a large and representative set of neurons. This tool is freely available upon request.

To implement and test our algorithms, we used digitally reconstructed hippocampal neurons from different repositories, grouped as follows: Group 1: 6 Dentate Gyrus 'aged' Granule Cells (referenced as n270-n275 from the Duke-Southampton archive; http://neuron.duke.edu), Group 2: 4 CA3 'young' Pyramidal Cells (referenced as l10, 148a, 160b and 164 from the Duke-Southampton archive), Group 3: 15 CA1 'aged' Pyramidal Cells (referenced as n170-n184 from the Duke-Southampton archive), and Group 4: 18 CA1 'young' Pyramidal Cells (referenced as a series of pyramidal cells from the Gulyás CA1 repository; http://www.koki.hu/~gulyas/).

Our software imports the cells, which are stored in a non-proprietary *.swc format including a header of comments where each line is preceded by a '#' sign. These lines describe the program used for neuron tracing (usually Neurolucida, MicroBrightField, Inc.), localization of the neuron (region, Field/Layer, etc.), type of cell, contributor, reference, soma area, shrinkage correction, number and date versions. Followed by these remarks, the *.swc file consists of a [*n x 7*] matrix which contains the following fields: Index (Column 1), a user defined flag denoting the specific part of the structure (cell body, apical dendrites, basilar dendrites and axon; Column 2), 3D coordinates (x, y, z, in μm; Columns 3–5), radius (r, in μm; Column 6), and parent index (Column 7). As two points connected by a straight line constitute a segment, then each neuronal reconstruction with *n* points has *n-1* total segments, where *n* is the maximum row size of the matrix. The import file function deletes the header and stores the coordinates into a [*n x 7*] matrix in MATLAB’s workspace for further analysis. In all cases the 'x', 'y' and 'z' values were corrected for shrinkage and lens medium refraction.

**Figure 1:**
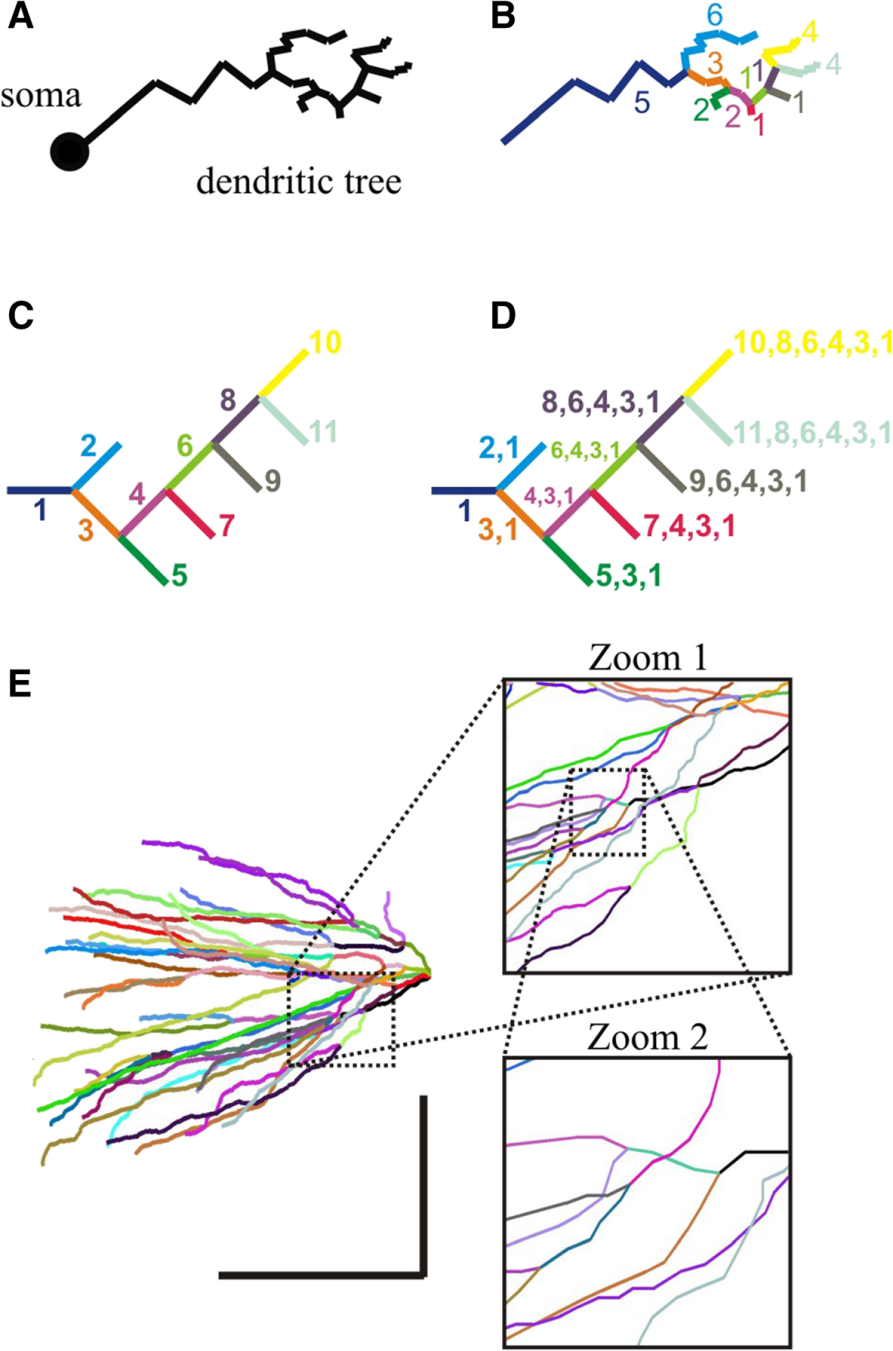
**Branch identification and genealogy. (A)** According to a putative digital neuronal reconstruction, it is possible to isolate all segments that belong to a specific dendritic tree. **(B)** The branch identification procedure allows detecting all branches (represented with different colors), each one containing a series of connected segments (numbers of segments beside each branch). **(C)** First-name assignment provides an arbitrary but unique numerical label to each branch. **(D)** For a specific branch, full-name assignment 'routes' the names of the preceding branches backwards to the soma. A branch is identified by its full-name, which also provides information about its ancestors (*i.e.* how many ancestors it has and which are their first-names). **(E)** An isolated basal dendritic tree from CA1 cell pc4c_b (same as in Figure 2 , panel **E**). Different branches are rendered using different random-assigned colors. Calibration bars 100 μm.

The identification of the branches was performed following a series of rules that determine whether two segments belong to the same branch. A segment is specified as a pair of points joined by a straight line and defined in the matrix as a pair of rows where the parent index of the first row (column 7) equals the index of the second row (column 1). Graphically this is the line that connects (x_*i*_,y_*i*_,z_*i*_) with (x_*j*_,y_*j*_,z_*j*_) where '*i*' is the parent index and '*j*' is the index. A branch is therefore conformed by one or more segments. Two connected segments belong to the same branch only if: i) the parent index of the second segment equals the index of the first segment, ii) both segments share the same structure flag stored in column 2 and iii) there is no other segment in the whole matrix that has the same parent index. If these restrictions applied, a sequential numerical label was assigned to the two segments in order to specify that all segments with the same name belonged to the same branch. At the end of this procedure, the set of rows that share the same first-name conform a branch whose first-name is a unique numerical label (Figure 1A-C). Once each branch has its own first-name it is possible to track the path of its ancestors back to the soma (Figure 1D). Consequently, the 'full-name' of a branch is computed as follows: the parent index in each row points to another row, which belongs to a branch with an already assigned first-name. Unless both rows belong to the same branch (*i.e.* having the same first-name), this name corresponds to the row’s parent name. The row’s parent-name (*i.e.* a full name) is stored and this process is repeated until the whole branching genealogy is generated. Thus, at the end of this process, each row has an associated full-name which can be traced backwards to its ancestors (Figure 1D). Here again, the set of rows that share the same full-name conform a branch. Full-name length (number of ancestors+1) equals the path-length and this measure was further used to compute topographic measurements (see below). Our algorithm successfully identified branches and assigned a full-name to each of them. Plots assigning a pseudo-random color to each branch confirmed that all branches were correctly identified, including those that generated dichotomous bifurcations (Figure 1E). Additionally, the plotter was able to represent vector’s width equal to segment’s diameter and/or assign four different colors depending on the specific cell structure (cell body, apical dendrites, basal dendrites or axon) according to the user-defined flag stored in the second column of the matrix (not illustrated).

**Figure 2:**
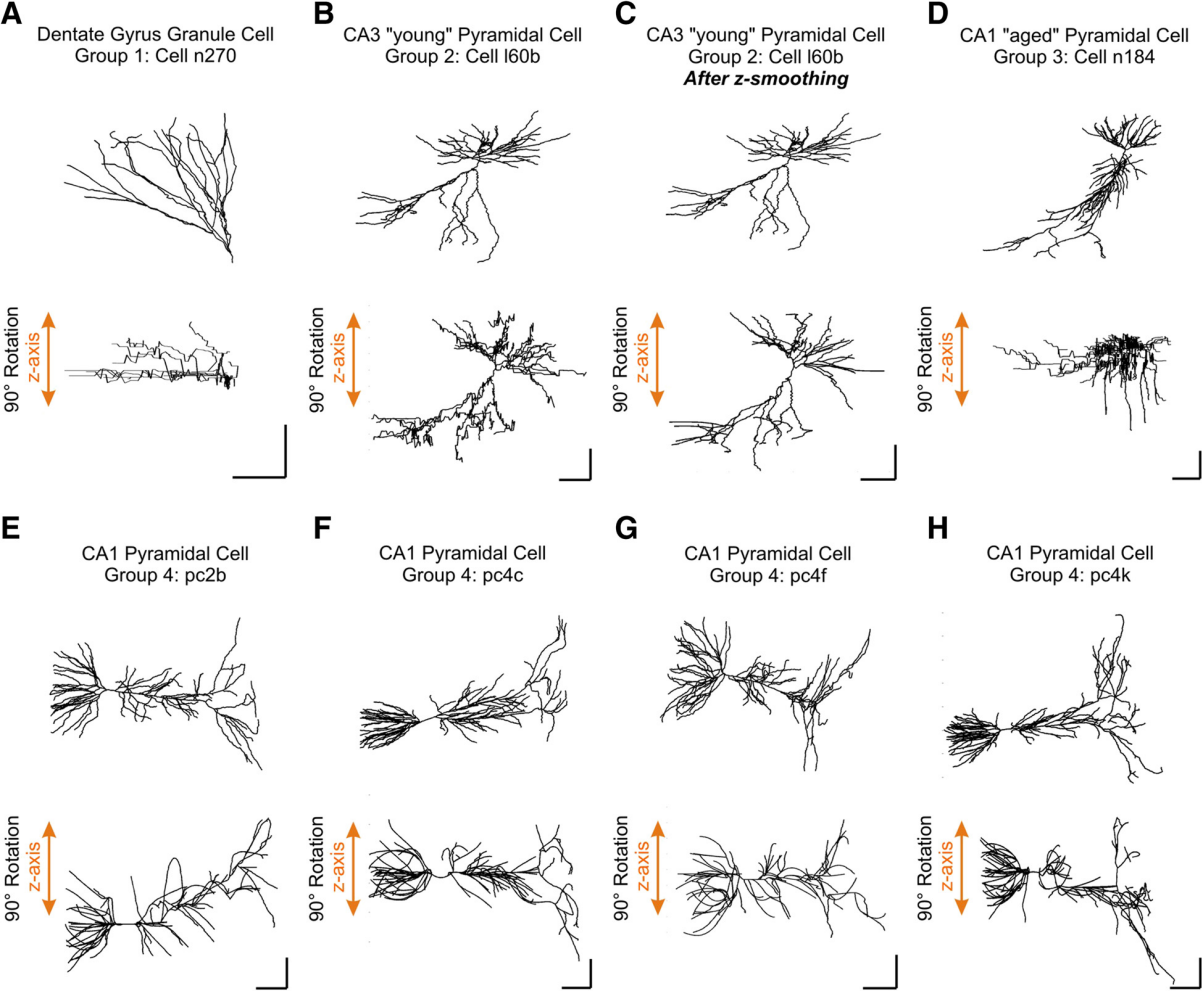
**Qualitative Neuronal Inspection.** The figure shows eight panels **(A**-**H)** rendering cells from the Duke-Southampton archive and the Gulyás repository (see Results and Discussion). Each panel presents one plotted cell seen from two different viewpoints. Top panels correspond to the 'xy' viewpoint and bottom panels to the 'yz' viewpoint (*i.e.* 90° rotation). Cells belonging to the Duke-Southampton archive have a similar z-axis noise [8]. Cell smoothing successfully abolishes z-noise (compare B vs. C, 'yz' viewpoint). Calibration bars 100 μm.

Before being subjected to quantitative analysis, we first plotted the experimentally reconstructed neuronal structures by computer-assisted graphical rendering, zooming, panning, and rotating (Figure 2). This allowed us to visualize the neurons as a series of black connected vectors (following index/parent index rules). Dendrites of hippocampal principal neurons displayed a polarized shape, as if they were enclosed in cones [5-7]. Furthermore, in hippocampal pyramidal cells (Figure 2B-H), basal and apical trees invaded opposite hemi-spaces, whereas the hippocampal granule cell tree invaded the same hemispace (Figure 2A). The dendritic arbors were clearly fanned out, each seemingly maintaining a preferred orientation throughout their length [6,7].

The analyzed cells from the Duke-Southampton archive exhibited a tendency to deviate repeatedly from the straight direction and return to the initial orientation after considerable meandering ('zigzags' towards the z-axis, Figure 2A, B). It is known that the acquisition and assembly procedures introduce morphological noise in any representation of digitized neurons [9-11], which makes it difficult to carry out a meaningful statistical analysis. We implemented a feature to perform z-coordinate smoothing aimed to diminish morphological parameter miscalculations due to such extensive amount of noise in the 'z' axis (Figure 2B, C). Specifically, this function smoothes the 'z' data of each branch using a moving average filter. As the spatial distribution of points defining segments is not uniform, their distance projected in the 'xy' plane is used as predictor data for the z-smoothing. However, applying this procedure to each independent branch would result in local 'z-jumps' at bifurcations and spatial continuity between related branches is required. To solve this problem, the smoothing was performed concatenating two additional points located at the branch’s endings: the first point given by an average of the final z-coordinate of the parent plus the initial z-coordinate of the sister. The latter represents the average of the initial z-coordinates of all daughters (if present). Aside from these issues, it is important to mention that the criteria for sampling data points for a morphological structure are subjective and to some extent arbitrary. Due to the complexity of dendritic morphology, the very same neurons mounted on microscope slides, and traced by different researchers or on different reconstruction systems, can result in considerably different digital files [10]. In this context, the issue of quality control for morphological data is extremely important and should be taken carefully into consideration in any morphological study before interpreting the results. In other words, digital files of dendritic morphology are rarely accurate representations of biological structures; they constitute only an approximation of the neuron. Nevertheless, if a digital data set is internally consistent (*v.gr.* correct indexing, no '0-length segments', etc.), then the mathematical problem of its quantitative representation is independent on the data quality.

**Figure 3:**
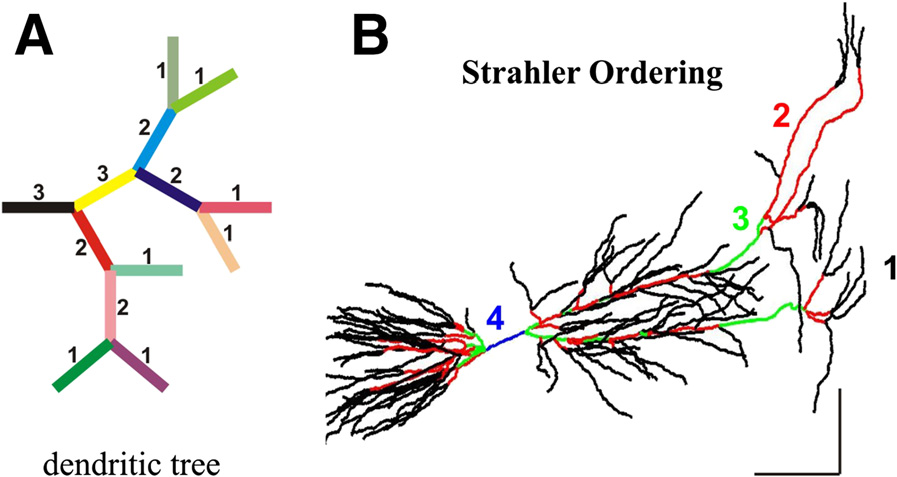
**Strahler ordering scheme. (A)** Schematic drawing of a dendritic tree with full-named branches. Computation of counters corresponding to the Strahler ordering for each branch. **(B)** Strahler Ordering Scheme for a reconstructed cell. A pyramidal CA1 cell from the Gulyás repository (Group 4, pc4c) was used to validate an effective Strahler indexing. The plot shows each branch with a different color (black, red, green and blue) corresponding to Strahler indexes (1, 2, 3 and 4) respectively. Notice the high number of terminal branches at the proximal and distal apical dendritic tree.

Geographical studies have proposed various ways of ordering branches in a stream network. Contemporary interest in stream ordering derives largely from the work of Horton [12], who drew attention to a number of empirical regularities, usually now known as Horton’s laws. His ordering scheme, however, is described as a variant of a method proposed later by Strahler [13]. 'Strahler ordering' assigns a number to each segment of the tree, which we refer to as the Strahler order number. Strahler ordering consists of the following steps: it starts with the ending branches of the rooted tree, that is, with those nodes, excluding the root, which are contiguous to only one edge. All edges that are contiguous to a branch are branches of order 1 (Figure 3A). These are streams with no tributary. When two or more branches of order *m* come together, the third edge contiguous to that branch belongs to a branch of order [*m+1*]. When a branch of order *m* meets a branch of order *n*, where *n* exceeds *m*, the third edge at the branch is a continuation of the branch of order *n* (Figure 3A). There are no arbitrary decisions in Strahler ordering, and it is purely topological. We tested the Strahler ordering function for correct indexing with a reconstructed neuron. Interestingly, all the pyramidal CA1 cells from the Gulyás repository had very low maximum Strahler indexes. This can be explained by the high number of terminal branches along their paths (Figure 3B).

Because cells belonging to the Gulyás repository (Group 4; Figure 2D-G) showed no z-noise, we used them for further testing of morphological analyses. Accumulative morphological measurement consists of those parameters that require calculations made by using information from multiple segments. For example, the length of a branch is computed as the sum of the Euclidean lengths (in 3D) from all segments belonging to this branch. Tapering, measures changes in diameter along all the segments within a branch. This is reported as the slope of a linear regression model adjusted to all diameter values as a function of the accumulative branch’s length. Additionally, a mean diameter/branch ± S.E.M is reported. Also, the distance metric (DM) tortuosity (a dimensionless number) provides a ratio between the actual path length of a meandering curve (*i.e.* along segments) and the linear distance between endpoints, whereas the sum of angles metric (SOAM) tortuosity integrates total curvature along a curve and normalizes it by path length [14,15], thereby handling tight coils better. Thus, for any point P_k_ we defined the vectors T_1_ = P_k_-P_k-1_, T_2_ = P_k+1_-P_k_, and T_3_ = P_k+2_-P_k+1_. The in-plane angle at point P_k_ (IP_k_) and the torsional angle (TP_k_) were given by the following equations, where TP_k_, IP_k_ ∈ [0,π]:

$$\begin{array}{l} \;IP_{k}=\cos^{-1}\left(\left(\frac{T1}{\left|T1\right|}\right)•\left(\frac{T2}{\left|T2\right|}\right)\right)\\ TP_{k}=\cos^{-1}\left(\left(\frac{T1\times T2}{\left|T1\times T2\right|}\right)•\left(\frac{T2\times T3}{\left|T2\times T3\right|}\right)\right) \end{array}$$

The total angle CP_k_ at point P_k_ is then

$$CP_{k}=\sqrt{\left(IP_{k}\times IP_{k}\right)+\left(TP_{k}\times TP_{k}\right)}$$

The SOAM calculates the total tortuosity of the curve as

$$\text{SOAM}=\frac{\overset{n-3}{\underset{k=1}{\sum }}CP_{k}}{\overset{n-1}{\underset{k=1}{\sum }}\left|P_{k}-P_{k-1}\right|}$$

Note that this expression normalizes the total curvature with respect to total curve length. This means that SOAM values can be compared between two branches of different length.

Bifurcation angles are taken as the angle between a daughter and its parent branch. To compute bifurcation angles between a daughter and its parent branch it is first necessary to calculate directional vectors at the beginning and at the end of each branch. The bifurcation angle between two branches does not only depend on its terminal segments. For this reason, when computing directional vectors it is necessary to consider several segments and then proceed with a 3D linear regression from where a normalized directional vector will be extracted. We used five segments per branch for directional vector computation (although we believe that the number of segments used should be justified in terms of their tortuosity).

Once accumulative morphological parameters are stored in the last row of each branch, it is possible to make a 'vertical compression' to produce a new matrix with each row corresponding to information from a branch with its respective accumulative morphological parameters.

Rall’s pioneering development of the idealized equivalent cylinder model for passive dendrites [16] opened up the field of neuronal modeling. One prediction of his model is that the sum of the diameters of the daughter branches at each bifurcation, raised to the 3/2 power, must equal the parent branch diameter also raised to 3/2 power (the '3/2 power rule'). Therefore, our software computes 'Rall’s exponent' for each particular branch point by minimizing the following difference:

deqXj-∑i=1ndiXj1R_ExponentR_Exponent=0

**Figure 4:**
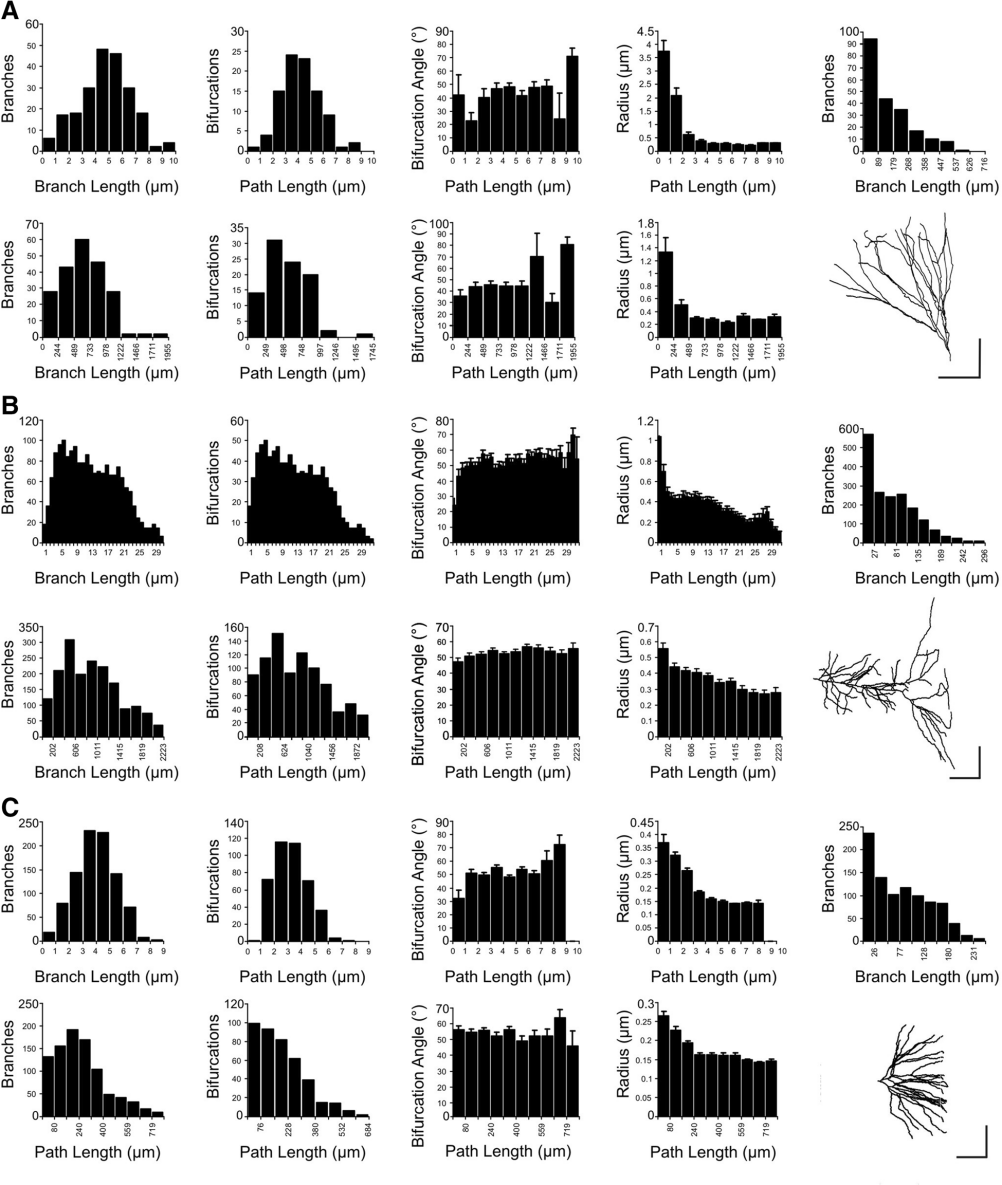
**Frequency Distributions of Morphological Parameters.** Three groups of dendrites from granule cells **(A)**, and from apical **(B)** and basal **(C)** dendrites of CA1 pyramidal cells were used to compute relevant morphological parameters such as number of branches, number of bifurcations, bifurcation angle, radius and number of branches with a specific branch length. Bin selection was made independently for each group according to Sturge’s rule. Bar-plots represent mean ± standard error of the mean. Calibration bars 100 μm.

**Figure 5:**
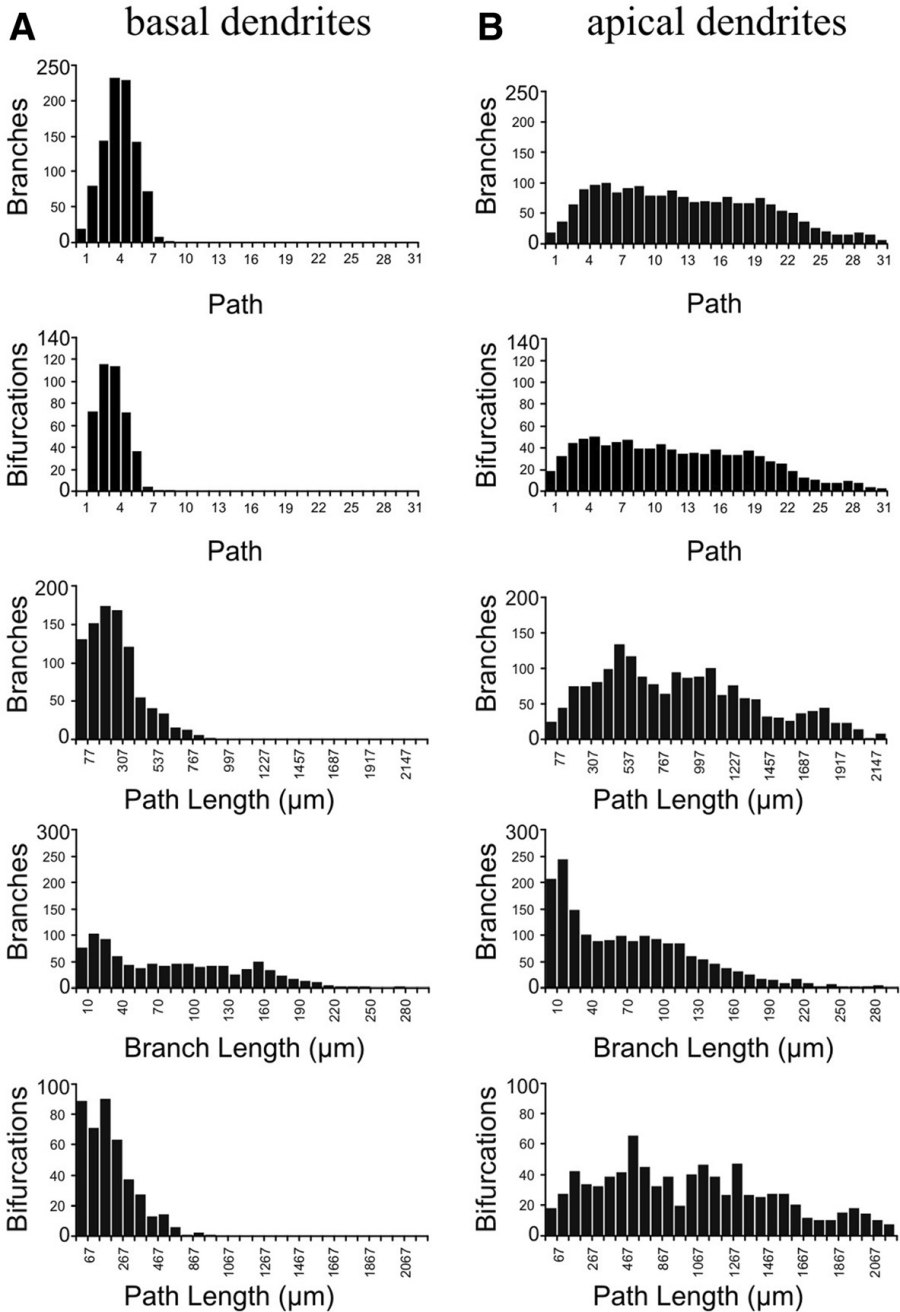
**Formal Comparison between Apical and Basilar Dendritic Trees.** Data from basal **(A)** and apical **(B)** dendrites from CA1 pyramidal cells were grouped and frequency distributions were computed for different morphological parameters using the same bin width. Apical CA1 dendritic trees project at longer distances than basilar dendritic trees. This is also evident when comparing the number of branches and bifurcations at different paths from the soma (two upper panels). Interestingly, branches from both, apical and basal dendritic trees show a similar shape for the branch length distributions. This suggests that both dendritic trees are constructed upon equally long branches (panels in the fourth row). Thus, a longer tree is a consequence of an increased number of total branches.

All basic parameters were measured from digital files of traced neurons. Raw data for each parameter were extracted in the form of simple arrays, grouped for each cell class and characterized with histograms representing frequency distributions. Group results for three groups of granule cells (4A), apical (4B), and basilar (4C) dendritic trees of CA1 pyramidal cells are shown in Figure 4. Bin selection was made independently for each group according to Sturge’s rule. However, selecting the same bin width for each histogram distribution allowed us to detect correlations between measurements for the same trees (such as depth×length and Euclidean distance×path distance; data not shown) as well as differences between morphological basic parameters of different dendritic trees (Figure 5). For example, apical CA1 dendritic trees project at longer distances than basilar dendritic trees. This feature is reflected in the frequency distribution of the number of branches located at different 'Euclidean' path lengths (with the starting point at the soma), and also when comparing the number of branches at different paths from the soma (Figure 5). Notably, branches from both dendritic trees show similar lengths, which indicates that both dendritic trees are constructed upon equally long branches (Figure 5). In other words, a longer dendritic tree (in spatial terms) is a consequence of the number of total branches employed to build the tree.

Elucidating the complex organization of the brain will require synthesis of information about neuron types, the spatial patterns of their dendritic and axonal arborizations, cell numbers and densities, as well as synapse number and location [17,18]. In the central nervous system, the shape of the dendritic arbor is related to the cell-type specificity and to the large number of synaptic inputs. The extent of dendritic arbors, at least in sensory neurons of the peripheral nervous system, physically defines their receptive fields [19], and axonal topology is known to affect synaptic output [20]. Discoveries that many dendrites conduct input signals actively, back-propagate action potentials, and integrate synaptic inputs by means of time-dependent nonlinear summation provide indisputable evidence that dendritic morphology is a key aspect of the neuronal machinery underlying signal processing and integration [21]. Dendritic structure contributes significantly to neuronal information processing [22,23] and computational models have shown that dendritic geometry can be responsible for producing an entire spectrum of firing patterns displayed across different cortical neuronal types [24], and also within a single class of hippocampal neurons [25]. The importance of dendrites for neuronal activity is evidenced by the influence of dendritic morphology on network connectivity [26] as it is constantly reshaped by the dynamic remodeling of both dendrites and axons, which is crucial in determining the pattern of synaptic formation among neurons [27].

Here, we developed a prototype of a MATLAB based software package to characterize neuronal dendrites on the basis of the statistical distributions of morphological parameters. From a merely morphological point of view and assuming that cells located on a specific site and under strict experimental conditions share similar morphological properties, the neuroanatomy of a cell class can be measured and compressed by quantifying statistical distributions of relevant morphological parameters. Such an approach is important for understanding the heterogeneity of the different neuronal groups, as well as for unveiling the relationship between neuronal structure and function. Hence, this tool can be applied for comparative anatomy, developmental neurobiology and medical diagnosis [4]. The resulting statistical descriptions of neuronal morphology can be further used to create an unlimited number of non-identical virtual neurons (data not shown). Virtual generation of axonal and dendritic arbors is useful to explore mechanisms of growth [28,29] and to construct biologically realistic neural networks [28,30].

## Competing interests

The authors have no financial competing interests.

## Authors’ contributions

MT: conceived ideas, wrote the software in MATLAB 7.8 (MathWorks, Inc.; Natick, USA and drafted the manuscript. All authors equally contributed analyzing data, making figures, writing and revising the manuscript. All authors read and approved the final version of the manuscript.

## References

[B1] Ascoli GA Mobilizing the base of neuroscience data: the case of neuronal morphologies Nat Rev Neurosci 2006 7 318 324 10.1038/nrn1885 16552417

[B2] Halavi M Hamilton KA Parekh R Ascoli GA Digital reconstructions of neuronal morphology: three decades of research trends Front Neurosci 2012 6 49 22536169 10.3389/fnins.2012.00049 PMC3332236

[B3] Meijering E Jacob M Sarria JC Steiner P Hirling H Unser M Design and validation of a tool for neurite tracing and analysis in fluorescence microscopy images Cytometry A 2004 58 167 176 15057970 10.1002/cyto.a.20022

[B4] Costa LF Zawadzki K Miazaki M Viana MP Taraskin S Unveiling the neuromorphological space Front Comput Neurosci 2010 4 150 21160547 10.3389/fncom.2010.00150 PMC3001740

[B5] Bradke F Dotti CG Establishment of neuronal polarity: lessons from cultured hippocampal neurons Curr Opin Neurobiol 2000 10 574 581 10.1016/S0959-4388(00)00124-0 11084319

[B6] Trevino M Vivar C Gutierrez R Beta/gamma oscillatory activity in the CA3 hippocampal area is depressed by aberrant GABAergic transmission from the dentate gyrus after seizures J Neurosci 2007 27 251 259 10.1523/JNEUROSCI.3815-06.2007 17202493 PMC6672272

[B7] Trevino M Vivar C Gutierrez R Excitation-inhibition balance in the CA3 network–neuronal specificity and activity-dependent plasticity Eur J Neurosci 2011 33 1771 1785 10.1111/j.1460-9568.2011.07670.x 21501253

[B8] Kulik A Vida I Lujan R Haas CA Lopez-Bendito G Shigemoto R Subcellular localization of metabotropic GABA(B) receptor subunits GABA(B1a/b) and GABA(B2) in the rat hippocampus J Neurosci 2003 23 11026 11035 14657159 10.1523/JNEUROSCI.23-35-11026.2003 PMC6741037

[B9] Horcholle-Bossavit G Gogan P Ivanov Y Korogod S Tyc-Dumont S The problem of the morphological noise in reconstructed dendritic arborizations J Neurosci Methods 2000 95 83 93 10.1016/S0165-0270(99)00159-4 10776818

[B10] Kaspirzhny AV Gogan P Horcholle-Bossavit G Tyc-Dumont S Neuronal morphology data bases: morphological noise and assesment of data quality Network 2002 13 357 380 10.1088/0954-898X/13/3/307 12222819

[B11] Hamam BN Kennedy TE Visualization of the dendritic arbor of neurons in intact 500 microm thick brain slices J Neurosci Methods 2003 123 61 67 10.1016/S0165-0270(02)00341-2 12581850

[B12] Horton RE Erosional development of streams and their drainage basins Bull Geological Soc Am 1945 56 275 370 10.1130/0016-7606(1945)56[275:EDOSAT]2.0.CO;2

[B13] Strahler AN Hypsometric analysis of erosional topography Bull Geol Soc Am 1952 63 1117 1142 10.1130/0016-7606(1952)63[1117:HAAOET]2.0.CO;2

[B14] Bullitt E Gerig G Pizer SM Lin W Aylward SR Measuring tortuosity of the intracerebral vasculature from MRA images IEEE Trans Med Imaging 2003 22 1163 1171 10.1109/TMI.2003.816964 12956271 PMC2430603

[B15] Hart WE Goldbaum M Cote B Kube P Nelson MR Measurement and classification of retinal vascular tortuosity Int J Med Inform 1999 53 239 252 10.1016/S1386-5056(98)00163-4 10193892

[B16] Rall W Branching dendritic trees and motoneuron membrane resistivity Exp Neurol 1959 1 491 527 10.1016/0014-4886(59)90046-9 14435979

[B17] DeFelipe J From the connectome to the synaptome: an epic love story Science 2010 330 1198 1201 10.1126/science.1193378 21109663

[B18] Parekh R Ascoli GA Neuronal morphology goes digital: a research hub for cellular and system neuroscience Neuron 2013 77 1017 1038 10.1016/j.neuron.2013.03.008 23522039 PMC3653619

[B19] Hall DH Treinin M How does morphology relate to function in sensory arbors? Trends Neurosci 2011 34 443 451 10.1016/j.tins.2011.07.004 21840610 PMC3166259

[B20] Sasaki T Matsuki N Ikegaya Y Effects of axonal topology on the somatic modulation of synaptic outputs J Neurosci 2012 32 2868 2876 10.1523/JNEUROSCI.5365-11.2012 22357869 PMC6621900

[B21] Eilers J Konnerth A Dendritic signal integration Curr Opin Neurobiol 1997 7 385 390 10.1016/S0959-4388(97)80067-0 9232799

[B22] Mel BW Ruderman DL Archie KA Translation-invariant orientation tuning in visual "complex" cells could derive from intradendritic computations J Neurosci 1998 18 4325 4334 9592109 10.1523/JNEUROSCI.18-11-04325.1998 PMC6792789

[B23] Stuart G Spruston N Sakmann B Hausser M Action potential initiation and backpropagation in neurons of the mammalian CNS Trends Neurosci 1997 20 125 131 10.1016/S0166-2236(96)10075-8 9061867

[B24] Mainen ZF Sejnowski TJ Influence of dendritic structure on firing pattern in model neocortical neurons Nature 1996 382 363 366 10.1038/382363a0 8684467

[B25] Krichmar JL Nasuto SJ Scorcioni R Washington SD Ascoli GA Effects of dendritic morphology on CA3 pyramidal cell electrophysiology: a simulation study Brain Res 2002 941 11 28 10.1016/S0006-8993(02)02488-5 12031543

[B26] van Ooyen A Duijnhouwer J Remme MW van Pelt J The effect of dendritic topology on firing patterns in model neurons Network 2002 13 311 325 10.1088/0954-898X/13/3/304 12222816

[B27] Wong WT Wong RO Rapid dendritic movements during synapse formation and rearrangement Curr Opin Neurobiol 2000 10 118 124 10.1016/S0959-4388(99)00059-8 10679440

[B28] Eberhard JP Wanner A Wittum G NeuGen: a tool for the generation of realistic morphology of cortical neurons and neural networks in 3D Neurocomputing 2006 70 327 342 10.1016/j.neucom.2006.01.028

[B29] van Ooyen A Using theoretical models to analyse neural development Nat Rev Neurosci 2011 12 311 326 10.1038/nrn3031 21587288

[B30] Koene RA Tijms B van Hees P Postma F de Ridder A Ramakers GJ NETMORPH: a framework for the stochastic generation of large scale neuronal networks with realistic neuron morphologies Neuroinformatics 2009 7 195 210 10.1007/s12021-009-9052-3 19672726

